# Effects of two different types of luteal support on pregnancy outcomes following antagonist fresh embryo transfer: a retrospective study

**DOI:** 10.1186/s12884-023-05570-0

**Published:** 2023-05-04

**Authors:** Minji De, Lixue Chen, Lin Zeng, Yang Wang, Rui Yang, Rong Li, Hongbin Chi

**Affiliations:** 1 Reproductive Medicine Center, Peking University Third Hospital, No. 49, North Garden Road, Haidian District, Beijing, 100191 China; 2 Department of Obstetrics and Gynaecology, Ewenki People’s Hospital, Hulunbuir, 021100 China; 3 Clinical Epidemiology Research Center, Peking University Third Hospital, No.49, North Garden Road, Haidian District, Beijing, 100191 China

**Keywords:** In vitro fertilization-embryo transfer, Antagonists, Luteal support, Pregnancy outcome, Propensity score matching

## Abstract

**Background:**

Only a small number of studies have reported the use of progesterone vaginal gel in combination with dydrogesterone as part of the antagonist protocol for fresh embryo transfer. Therefore, this study aimed to compare the effects of two types of luteal support on pregnancy outcomes following the antagonist protocol for fresh embryo transfer.

**Methods:**

We performed a retrospective analysis of clinical data from infertile patients who underwent fresh embryo transfer via the antagonist protocol (2785 cycles) between February and July 2019 and between February and July 2021 at the Peking University Third Hospital Reproductive Medicine Centre. According to the luteal support received, the cycle groups were divided into the progesterone vaginal gel group (single medication or VP group; 1170 cycles) and the progesterone vaginal gel plus dydrogesterone group (combination medication or DYD + VP group; 1615 cycles). After propensity score matching, the clinical pregnancy, ongoing pregnancy, early miscarriage, and ectopic pregnancy rates were compared between the two groups.

**Results:**

In total, 1057 pairs of cycles were successfully matched via propensity scores. The clinical and ongoing pregnancy rates in the combination medication group were significantly higher than those in the single medication group (*P* < 0.05), whereas no significant differences were noted in the early miscarriage and ectopic pregnancy rates between the two groups (both *P* > 0.05).

**Conclusions:**

Combined luteal support after the antagonist protocol is preferred for patients undergoing fresh cycle embryo transfer.

## Background

Normal luteal function is an important factor for maintaining pregnancy; luteal phase insufficiency decreases embryo implantation and pregnancy rates and increases the early miscarriage rate. The prevalence of luteal insufficiency in the ovulation-promoting cycle population is 12%–20% [1], while that in the natural cycle population is only 3%–10% [2]. Therefore, luteal support is an essential component of in vitro fertilization-embryo transfer (IVF-ET). Several meta-analyses have confirmed that enhancing luteal support can significantly improve the pregnancy outcomes of IVF-ET [3–5]. The guidelines used for the maintenance of pregnancy with luteal support and progestogen also note that luteal support in the early luteal phase of human-assisted reproductive technology (ART) can improve the pregnancy outcomes [6]. While several protocols exist, to the best of our knowledge, no commonly accepted protocol has been established for optimal luteal support after IVF-ET to date, and whether drugs should be administered alone or in combination remains controversial. Only a few studies have reported the use of progesterone vaginal gel in combination with dydrogesterone in the antagonist protocol for fresh embryo transfer. Therefore, this study aimed to compare the effects of these two luteal support protocols on pregnancy outcomes after IVF-ET with the antagonist protocol to clarify a rational protocol of luteal support.

## Methods

### Study participants

**Fig. 1:**
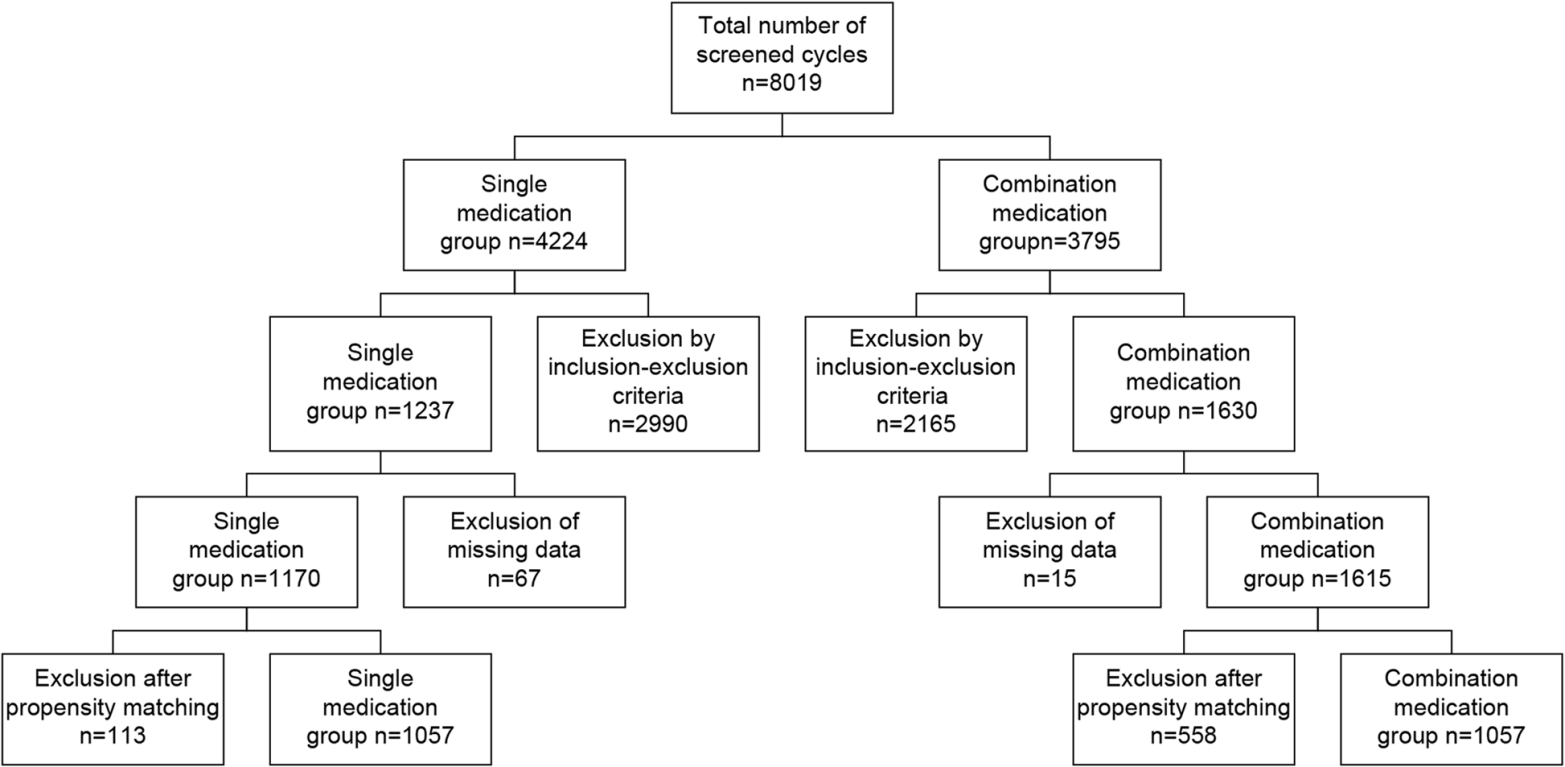
Data screening process diagram

In this retrospective study, clinical data of infertile patients who underwent fresh embryo transfer with the antagonist protocol at the Peking University Third Hospital Reproductive Medicine Centre from February to July 2019 and from February to July 2021 were obtained. Patients with the following criteria were screened for study inclusion: 1) being 20–45-year-old; 2) having primary or secondary infertility due to tubal factors, endometriosis, male factors, or unknown causes; 3) having undergone the gonadotropin-releasing hormone (GnRH)-antagonist (GnRH-ant) protocol; and 4) having received fresh embryo transfer. Patients with 1) an abnormal uterine environment owing to uterine adhesions, submucosal fibroids, adenomyosis, or congenital uterine abnormalities; 2) repeated implantation failure; 3) recurrent miscarriage; 4) tubal effusion; 5) chromosomal and genetic abnormalities in either spouse subjected to pre-implantation genetic testing; 6) immune abnormalities, such as systemic lupus erythematosus and antiphospholipid syndrome; 7) ≥ 3 cycles of transfer or failure of ovulation promotion; 8) intrauterine pregnancy combined with ectopic pregnancy or molar pregnancy; and 9) a trigger mode reduced to double trigger or GnRH**–** a single trigger**–** were excluded. A total of 2785 cycles meeting the criteria were included and divided into two groups according to the type of luteal support received: the single medication group (1170 cycles) and the combination medication group (1615 cycles) (Fig. 1).

### Ovulation promotion and embryo transfer

All patients were treated with the antagonist protocol. Gonadotropin (Gn) was injected on the 2nd day of the menstrual cycle, and transvaginal ultrasound was performed to detect follicle growth from the 4th day after the Gn injection. On the 7th to the 8th day of the menstrual cycle or when the follicle diameter was ≥ 14 mm, patients received injections of 0.25 mg GnRH-ant until the day of trigger. The Gn starting dose was between 75–450 IU, which was adjusted according to ultrasound monitoring of follicles and serum estradiol levels. Triggers were administered when at least two of the dominant follicles were ≥ 18 mm in diameter. An hCG trigger was administered according to the E2 level on the trigger day. After 34–36 h, oocytes were retrieved under vaginal ultrasound guidance for conventional IVF or intracytoplasmic sperm injection, and one–two embryos at the cleavage stage or one embryo at the blastocyst stage were transferred.

### Embryo and blastocyst evaluation criteria

The quality of D3 embryos was evaluated using Peter’s cleavage-stage embryo scoring system, in which embryos with ≥ 8 cells with grades I–II were considered high-quality embryos. Gardner’s grading standard was used to score blastocysts; blastocysts with stage ≥ 4 were considered high-quality blastocysts.

### Luteal support

The combination medication group received both progesterone vaginal gel (90 mg daily dose) and dydrogesterone oral capsules (10 mg twice a day) from the day of oocyte retrieval. If pregnancy was confirmed, luteal support was continued for 30 days after embryo transfer. For intrauterine pregnancy confirmed by vaginal ultrasound, luteal support was continued until approximately 7–8 weeks of intrauterine pregnancy, at which time the progesterone vaginal gel was discontinued. The dydrogesterone tablets were continued, with the dosage gradually reduced until 8–10 weeks of pregnancy, when luteal support was discontinued. In the single medication group, 90 mg of progesterone vaginal gel was administered daily. Luteal support for confirmed pregnancies was continued for 30 days following embryo transfer, while that for confirmed intrauterine pregnancies was continued until 8–10 weeks of pregnancy.

### Determination of pregnancy outcome

At 2 weeks after embryo transfer, serum was drawn to assess the β-hCG level, and an hCG level of ≥ 5 mIU/mL was considered a positive pregnancy test. Miscarriage, ectopic pregnancy, and pathological examination of chorionic villi were considered clinical pregnancies when observed on vaginal ultrasound at 30 days following the transfer. The presence of embryonic arrest or spontaneous miscarriage was regarded as miscarriage.

### Observation indicators

Basic patient data, including age, body mass index (BMI), years of infertility, type of infertility, and days of Gn medication, were compared between both groups. Pregnancy outcome indicators included clinical pregnancy, ongoing pregnancy, miscarriage, and ectopic pregnancy rates. The clinical pregnancy rate (CPR) was defined as the number of clinical pregnancies divided by the number of total transplantations. The ongoing pregnancy rate (OPR) was defined as the number of the presence of at least one fetus with fetal pulsation on ultrasound beyond 12 weeks divided by the number of total transplantation. The early miscarriage rate (EMR) was defined as the number of miscarriages that occurred before 12 weeks divided by the number of positive pregnancies. The ectopic pregnancy rate (EPR) was defined as the number of ectopic pregnancy cycles divided by the number of clinical pregnancies.

### Propensity score matching

The MatchIt package in R (R Foundation for Statistical Computing, Vienna, Austria) was used to match the two groups of patients by the propensity score matching method, with the grouping situation as the dependent variable, and age, basal follicle-stimulating hormone (FSH), anti-Müllerian hormone (AMH), endometrial thickness on the trigger day, number of embryos transferred, type of embryos transferred, trigger mode, and number of oocytes obtained as covariates. Propensity scores were calculated by logistic regression, and the 1:1 nearest neighbor matching method was used, with the caliper value set at 0.2 to ensure excellent matching results.

### Statistical methods

Statistical analyses were performed using SPSS 26.0 (IBM Corp., Armonk, NY, USA) and R4.03 statistical software. Covariate equilibrium tests were performed on the matched samples between the groups. Statistical analyses were performed using newly matched samples. Measurement data for the normal distribution variables are expressed as means ± standard deviations, and comparisons between the two groups were conducted using an independent-samples *t*‑test. The variables in line with non‑normal distribution are expressed as the median (p25, p75) and were compared using the rank-sum test. Numerical data are expressed as n (%) and were compared using the Chi‑square test. A logistic regression model was used for multivariate analysis. The difference was considered statistically significant at *P* < 0.05 (two‑sided).

## Results

### Propensity score matching

Significant differences were noted in age, basal FSH and AMH levels, number of oocytes obtained, and trigger mode between the two groups, which were not comparable. Thus, the 1:1 nearest neighbor matching method was used to effectively improve the balance of variables between the non-randomized data groups to eliminate selection bias and reduce the effects of bias and confounding variables. The single and combination medication groups with complete data before matching comprised 1170 and 1615 cycles, respectively, and 1057 pairs of cycles were successfully matched between the two groups. After matching, all unbalanced covariates between the two groups reached equilibrium and were comparable (Table 1).

**Table 1:** Comparison of general information before and after data matching between the two groups

Parameter	Before matching			After matching		
	VP *n* = 1170	DYD + VP *n* = 1615	*P*-value	VP *n* = 1057	DYD + VP *n* = 1057	*P*-value
Age (years)	32.75 ± 4.54	33.10 ± 4.36	0.04	32.83 ± 4.59	32.83 ± 4.44	0.99
FSH (IU/L)	7.42 ± 2.76	6.57 ± 2.30	< 0.001	7.16 ± 2.53	6.99 ± 2.54	0.13
AMH	2.96 ± 2.64	2.65 ± 2.43	0.002	2.89 ± 2.63	2.78 ± 2.67	0.33
EM (mm)	10.67 ± 1.59	10.64 ± 1.66	0.74	10.67 ± 1.59	10.65 ± 1.63	0.86
Number of embryos transferred			0.53			0.82
1	201 (17.2%)	263 (16.3%)		184 (17.4%)	188 (17.8%)	
2	969 (82.8%)	1352 (83.7%)		873 (82.6%)	869 (82.2%)	
Number of oocytes obtained	9.60 ± 4.40	8.65 ± 3.99	< 0.001	9.36 ± 4.33	9.07 ± 4.17	0.12
Type of embryos transferred			0.05			0.48
D3	1126 (96.2%)	1575 (97.5%)		1016 (96.1%)	1022 (96.7%)	
D5	44 (3.8%)	40 (2.5%)		41 (3.9%)	35 (3.3%)	
Trigger mode			< 0.001			0.11
Single trigger	601 (51.4%)	1137 (70.4%)		592 (56%)	628 (59.4%)	
Double trigger	569 (48.6%)	478 (29.6%)		465 (44%)	429 (40.6%)	

*FSH* Follicle-stimulating hormone, *AMH* Anti-Müllerian hormone, *EM* Endometrial thickness on trigger day, *D3* day 3 embryo, *D5* day 5 embryo, *VP* Vaginal progesterone gel group (single medication group), *DYD* + *VP* dydrogesterone plus vaginal progesterone gel group (combination medication)

### Comparison of general information after data matching between the two groups

After matching, the differences between the two groups were not significant when comparing age, type of infertility, and years of infertility (all *P* > 0.05); however, BMI and days of Gn medication were significant (*P* < 0.05) (Table 2).

**Table 2:** Comparison of general information between the two groups after propensity score matching

Parameter	VP *n* = 1057	DYD + VP *n* = 1057	*P*-value
Age, in years			0.06
20–30	349 (33.02%)	323 (30.56%)	
31–35	414 (39.17%)	472 (44.65%)	
36–39	202 (19.11%)	170 (16.08%)	
≥ 40	92 (8.70%)	92 (8.71%)	
Type of infertility			0.19
Primary infertility	660 (62.44%)	689 (65.18%)	
Secondary infertility	397 (37.56%)	368 (34.82%)	
Years of infertility			0.50
< 5 years	867 (82.02%)	886 (83.82%)	
5–10 years	162 (15.33%)	143 (13.53%)	
≥ 10 years	28 (2.65%)	28 (2.65%)	
BMI	22.31 (20.43, 24.97)	22.03 (20.05, 24.47)	0.03
Days of Gn medication	10 (9, 11)	10 (9, 11)	0.02

*BMI* Body mass index, *Gn* gonadotropin, *VP* Vaginal progesterone gel group (single medication group), *DYD* + *VP* Dydrogesterone plus vaginal progesterone gel group (combination medication)

### Pregnancy outcomes

The clinical and ongoing pregnancy rates were significantly different between the combination and single medication groups (42.29% [*n* = 447] vs 36.80% [*n* = 389]; *P* = 0.01 and 35.57% [*n* = 376] vs 31.22% [*n* = 330]; *P* = 0.03, respectively). No significant differences were noted in the early abortion or ectopic pregnancy rates between the combination and single medication groups (13.2% [*n* = 59] vs 10.54% [*n* = 41] and 2.68% [*n* = 12] vs 4.63% [*n* = 18], respectively; all *P* > 0.05) (Table 3).

**Table 3:** Comparison of pregnancy outcomes between the groups

Parameter	DYD + VP *n* = 1057	VP *n* = 1057	*P*-value
CPR	42.29% (447)	36.80% (389)	0.01
OPR	35.57% (376)	31.22% (330)	0.03
EMR	13.20% (59)	10.54% (41)	0.24
EPR	2.68% (12)	4.63% (18)	0.13

*CPR* Clinical pregnancy rate, *OPR* Ongoing pregnancy rate, *EMR* Early miscarriage rate, *EPR* Ectopic pregnancy rate, *VP* Vaginal progesterone gel group (single medication group); *DYD* + *VP* Dydrogesterone plus vaginal progesterone gel group (combination medication)

### Logistic regression analysis

After matching, significant differences were noted in BMI and days of Gn medication between the two groups. Then, multifactor analysis was conducted to analyze the effect of lasso combined with medication on pregnancy outcomes. A multivariate logistic regression model was constructed with pregnancy outcome as the dependent variable and luteal support medication, BMI, and days of Gn medication as independent variables (Table 4). When BMI and Gn days were controlled, the OR values of the CPR, continuous pregnancy rate, early abortion rate, and EPR were 1.254 (1.053, 1.494; *P* = 0.01), 1.207 (1.007, 1.447; *P* = 0.04), 1.370 (0.892, 2.102; *P* = 0.15), and 0.551 (0.262, 1.165; *P* = 0.12), respectively, compared with the single medication group.

**Table 4:** Multi-variable Logistic regression of pregnancy outcomes between the groups

Outcome	OR	95%CI of OR	*P*-value
CPR	1.254	(1.053, 1.494)	0.01
OPR	1.207	(1.007, 1.447)	0.04
EMR	1.370	(0.892, 2.102)	0.15
EPR	0.551	(0.261, 1.165)	0.12

The logistic regression models were adjusted by BMI and days of Gn medication

## Discussion

Progesterone is the primary drug prescribed for luteal support. At present, various modes of administration and combinations of luteal support protocols are available as part of ART, such as intramuscular injection; oral, vaginal, or combination medication; intramuscular injection combined with oral; and others. Although the efficacy of intramuscular progestogen injection has been proven, it is gradually being replaced by oral and vaginal progesterone owing to its serious adverse effects. There are also different opinions concerning whether combination medication is better than single medication.

Patki et al. compared the effect of 600 mg micronized progesterone combined with 20 mg of dydrogesterone with 600 mg of micronized progesterone alone as luteal support for clinical pregnancy in a long protocol and found a higher CPR in the combination medication group than that in the single medication group [7]. This is consistent with our results. Tomic et al. compared the effect of 100 mg of micronized progesterone administered orally three times daily in combination with 90 mg of progesterone vaginal gel applied daily with that of 90 mg of progesterone vaginal gel applied daily on the pregnancy outcome of GnRH-a long protocol ovulatory fresh embryo transfer cycles. Interestingly, they found that the combination group had higher OPR and lower miscarriage rate than the single-treatment group [8], in consistency with our findings. Devine et al. showed that while the single medication group had the same embryo implantation rate as the combination medication group, the combination medication group had a higher OPR than that of the single medication group. They concluded that high local progesterone levels are sufficient to maintain embryo implantation and that higher and more stable serum progesterone levels are required for ongoing pregnancies [9]. This suggests the feasibility of using a combination of medications as luteal support.

Dydrogesterone is an orally administered reverse progesterone with an average bioavailability of 28%, which is 10–20 times higher than that of micronized progesterone capsules [10, 11] and does not alter the serum progesterone levels at its effective dosage of 10–20 mg/day. The advantages of dydrogesterone include its easy absorption, reduced hepatic load, convenience, good tolerability, high bioavailability, few side effects, and good compliance.

Progesterone vaginal gel is a new microfibrillated natural progesterone drug formulation with no hepatic first-pass effects that target the uterus and causes a “local high concentration effect” in the endometrium. Owing to the low absorption rate into the blood, its concentration in the blood is significantly lower, which effectively reduces the risk of systemic side effects. In addition, it can evoke a sedative effect on the uterus, increase the production of cervical mucus (thereby driving the formation of the cervical mucus plug), improve the Th1/Th2 balance, relax the uterine smooth muscle, significantly reduce the propagation of electrical signals in the myometrium, effectively reduce the frequency of uterine contractions [12], and reduce uterine arterial resistance [13], thus, increasing the pregnancy rate. A meta-analysis [14] comparing the efficacy of progesterone vaginal gel versus intramuscular progesterone for luteal support in assisted reproduction techniques suggested that both had good efficacy; however, the incidence of side effects associated with progesterone vaginal gel was lower than that with intramuscular progesterone, and the patients reported higher satisfaction with the progesterone vaginal gel than with intramuscular progesterone. It was also noted that the high incidence of early bleeding during the use of progesterone vaginal gel did not affect the pregnancy outcomes [15, 16], possibly because low serum progesterone levels do not effectively prevent short-term endometrial breakdown [17]. Therefore, a dynamic equilibrium between serum and local endometrial progesterone levels may be necessary to achieve good pregnancy outcomes, and topical vaginal medications combined with oral or intramuscular luteal support may be an important way to maintain this equilibrium.

In another previous study comparing progesterone vaginal gel with progesterone injection for luteal support in fresh embryo transfers as part of antagonist protocols, the application of progesterone vaginal gel resulted in better clinical outcomes than the progesterone injection [15]. Moreover, this study found that combination luteal support after an antagonist protocol resulted in higher clinical and ongoing pregnancy rates than those receiving a single luteal treatment, suggesting that enhanced luteal support is required for patients undergoing IVF-ET with the GnRH-ant protocol.

This study has some limitations. Especially, it was a single-center retrospective study with differences in baseline data between the two groups of patients. However, propensity score matching analysis was used to effectively improve the balance of covariates between groups and to eliminate selection bias between groups, such that the study results were more realistic.

## Conclusions

Progesterone vaginal gel combined with dydrogesterone luteal support provided better clinical outcomes than progesterone vaginal gel alone in antagonist protocol fresh cycle embryo transfer. Therefore, combined luteal support may be preferred for patients undergoing the antagonist protocol for fresh embryo transfer.

## Data Availability

The datasets used and/or analyzed during the current study are available from the corresponding author upon reasonable request.

## Abbreviations

CPR: Clinical pregnancy rate
OPR: Ongoing pregnancy rate
EMR: Early miscarriage rate
EPR: Ectopic pregnancy rate
IVF-ET: In vitro fertilization-Embryo transfer
ART: Assisted reproductive technology
GnRH-ant: Gonadotropin-releasing hormone- antagonist
GnRH-a: Gonadotropin-releasing hormone- agonist
hCG: Human chorionic gonadotropin
Gn: Gonadotropin
BMI: Body mass index
FSH: Follicle-stimulating hormone
AMH: Anti Müllerian hormone
